# An hTERT/ZEB1 complex directly regulates E-cadherin to promote epithelial-to-mesenchymal transition (EMT) in colorectal cancer

**DOI:** 10.18632/oncotarget.5968

**Published:** 2015-10-20

**Authors:** Yong Qin, Bo Tang, Chang-Jiang Hu, Yu-Feng Xiao, Rui Xie, Xin Yong, Yu-Yun Wu, Hui Dong, Shi-Ming Yang

**Affiliations:** ^1^ Department of Gastroenterology, Xinqiao Hospital, Third Military Medical University, Chongqing 400037, P.R. China

**Keywords:** hTERT, ZEB1, EMT, CRC

## Abstract

In human cancer, high telomerase expression is correlated with tumor aggressiveness and metastatic potential. Telomerase activation occurs through telomerase reverse transcriptase (hTERT) induction, which contributes to malignant transformation by stabilizing telomeres. Previous studies have shown that hTERT can promote tumor invasion and metastasis of gastric cancer, liver cancer and esophageal cancer. Epithelial-to-mesenchymal transition (EMT), a requirement for tumor invasion and metastasis, plays a key role in cancer progression. Although hTERT promotes EMT through Wnt signaling in several cancers, it is unknown if other signaling pathways are involved. In the present study, we found that hTERT and ZEB1 form a complex, which directly binds to the E-cadherin promoter, and then inhibits E-cadherin expression and promots EMT in colorectal cancer cells. hTERT overexpression in HCT116 and SW480 cells could induce E-cadherin down-regulation. However, E-cadherin expression was recovered when ZEB1 function was impaired even during hTERT overexpression. Taken together, our findings suggest that hTERT can promote cancer metastasis by stimulating EMT through the ZEB1 pathway and therefore inhibiting them may prevent cancer progression.

## INTRODUCTION

Colorectal cancer (CRC) is a major cause of cancer mortality in Western countries as it has a propensity to metastasize [1]. Although CRC has a low incidence in China, it is becoming the fastest growing cancer with the development of our society [2].

Telomerase is a reverse transcriptase that carries its own templates, and maintains telomere length by synthesizing telomeric DNA repeats. Therefore, telomerase maintains chromosome stability and enhances the ability of cells to promote cell immortality [3, 4]. The human telomerase reverse transcriptase (hTERT) is a reverse transcriptase that synthesizes telomeric DNA to maintain and increase telomere length. hTERT which is expressed only in immortalized cells and most tumor cells plays an important role in tumor occurrence and development [5].

Epithelial-to-mesenchymal transition (EMT) is a key factor that promotes malignant cell invasion into the surrounding tissues of most carcinomas [6]. The canonical Wnt pathway is an important signal that can initiate EMT by triggering β-catenin translocation to the nucleus, where it acts as coactivator of T-cell and lymphoid enhancer (TCF–LEF) factors in target gene transcriptional activation [7]. While hTERT can promote migration and invasion through the Wnt signaling pathway to induce EMT in gastric cancer [8], Wnt signal is not activated in some tumors, such as in CRC [9]. And further study found that HCT116 cells, a CRC cell line, manifested a low Wnt activity [10]. Therefore, hTERT may promote EMT in CRC via the mechanisms other than canonical Wnt pathway.

ZEB1 (zinc-finger E-box binding homeobox 1) is a key EMT nuclear transcription factor as it can bind to the E-cadherin promoter E-box region and has an important role in regulating E-cadherin expression [11, 12]. E-cadherin is an epithelial marker and an EMT initiating factor. Down-regulation, inhibition, or loss of E-cadherin can activate EMT [13]. Sanchez-Tillo previously showed that binding of ZEB1 to E-cadherin promoter requires Brg1 recruitment [14], we therefore sought to test if hTERT can combine with ZEB1, which then binds to E-cadherin promoter region, and ultimately inhibits its expression and promotes EMT.

In the present study, we found that hTERT interacts with ZEB1 to form a complex that directly binds to the −1959 bp to −1954 bp E-cadherin promoter region, represses E-cadherin expression to induce EMT, and finally promotes cell migration and invasion. This study reveals that hTERT promotes EMT through a novel pathway.

## RESULTS

### hTERT promotes EMT in CRC cell lines

**Figure 1:**
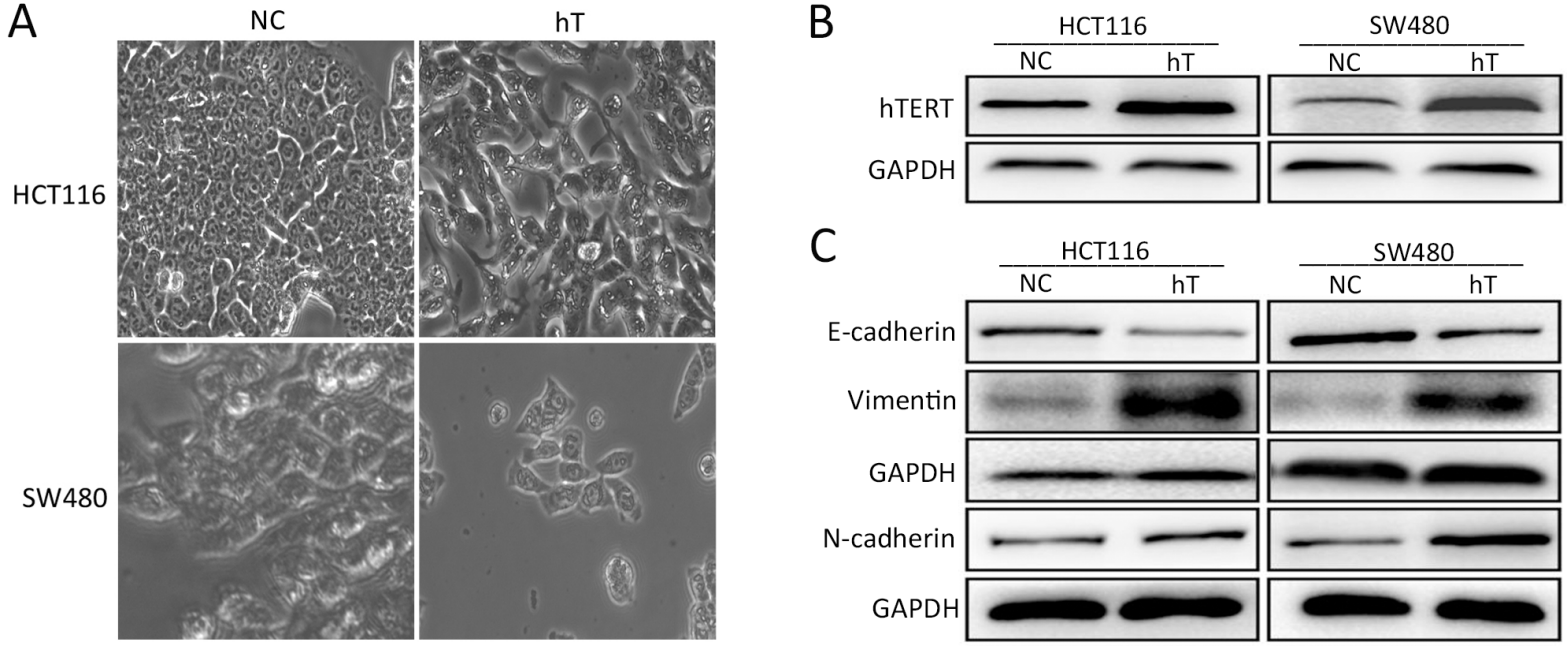
hTERT promotes EMT in colorectal cancer cell lines **A.** Morphological changes in SW480 and HCT116 cells after hTERT overexpression. **B.** hTERTexpression in SW480 and HCT116 cells as detected by Western blot. **C.** Western blot of E-cadherin, *N*-cadherin and vimentin in SW480 and HCT116 cells.

Although hTERT has been shown to promote EMT in osteosarcoma, ovarian cancer, and gastric cancer [8, 15, 16], it is not known if it promotes EMT in CRC. Therefore, we transfected an hTERT lentivirus vector into SW480 and HCT116 CRC cell lines and observed that cells expressing high levels of hTERT showed fibroblastoid-like alterations (Figure 1A and 1B). We next analyzed epithelial and mesenchymal marker expression in NC-HCT116 cells infected with hTERT overexpression vector or empty vector (Figure 1C). There was a great reduction in E-cadherin expression and an increase in *N*-cadherin and vimentin in hT-HCT116 cells compared to control cells (Figure 1C). We observed similar expression in SW480 cells (Figure 1C). Immunofluorescence also showed similar results in SW480 and HCT116 cells (Supplementary Figure S1A and S1B). Interestingly, we also detected the changes of other EMT-associated factors, such as the snail's expression was elevated when hTERT was overexpressed in SW480, but not in HCT116 cells (Supplementary Figure S1C). This suggests that hTERT can induce EMT in CRC cells.

### hTERT induces EMT independent of Wnt signaling

**Figure 2:**
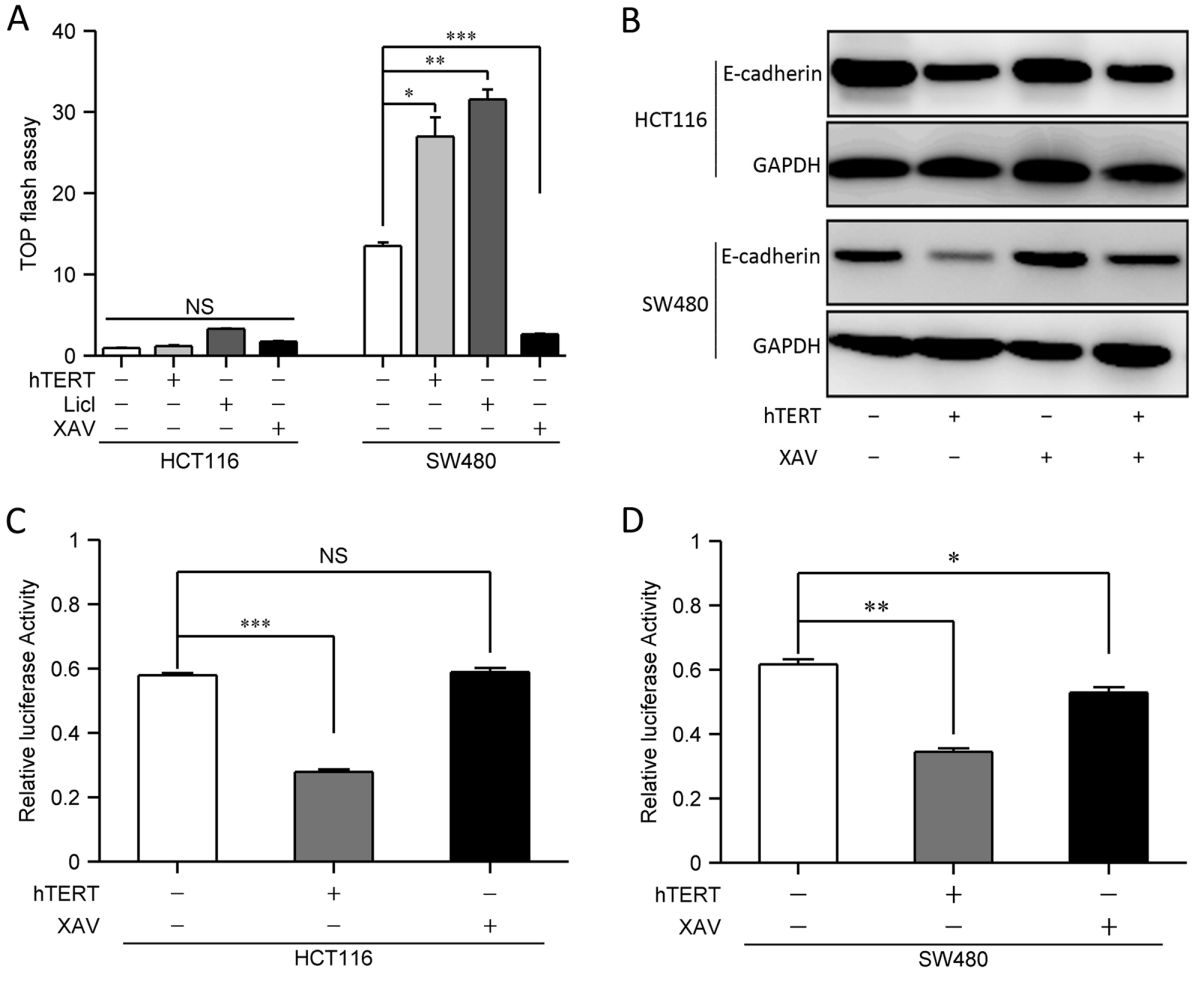
hTERTpromotes EMT independent of the Wnt signaling pathway **A.** Colorectal cancer cells were co-transfected with 100 nM hTERT plasmid and a TOP-FLASH reporter (0.1 ng/well in each 96-well plate), and Wnt signal agonists(LiCl) and inhibitor(XAV) were added TOP-FLASH reporter activation was tested 48 h later. A scrambled construct was used as a negative control (NC). *P < 0.05 compared to control. **B.** Western blot of E-cadherin in HCT116 and SW480 cells(Lane1, No treatment; Lane2, hTERT overexpression; Lane3, Wnt inhibition; Lane4, hTERT overexpression plus Wnt inhibition). **C–D.** Colorectal cancer cells were co-transfected with 100 nM hTERT plasmid and a luciferase reporter containing the E-cadherin promoter (0.1 ng/well in each 96-well plate), and luciferase activity was tested 48 h later. A scrambled construct was used as a negative control (NC). *P < 0.05 compared to control. (C) The relative luciferase activity of the E-cadherin promoter in HCT116 cells. (D) The relative luciferase activity of the E-cadherin promoter in SW480 cells.

Since previous studies showed that hTERT promotes EMT by activating the Wnt signaling in gastric cancer cells [8, 17], we examined Wnt activity in SW480 and HCT116 cells; however, we found that Wnt activity was significantly lower in HCT116 cells than in SW480 cells (Figure 2A). Further studies showed that hTERT could promote Wnt activity in SW480 cells, but not in HCT116 cells (Figure 2A). We also treated SW480 and HCT116 cells with a Wnt signaling activator (LiCl) and inhibitor (XAV), and found the expected responses in SW480 cells but not in HCT116 cells (Figure 2A). We next detected E-cadherin expression, which was significantly down-regulated both in SW480 and HCT116 cells by hTERT overexpression, and Wnt signal inhibition could restore E-cadherin expression in SW480 cells even when hTERT was overexpressed (Figure 2B and Supplementary Figure S2A). In contrast, Wnt inhibition could not restore E-cadherin expression in HCT116 cells (Figure 2B and Supplementary Figure S2B).

**Figure 3:**
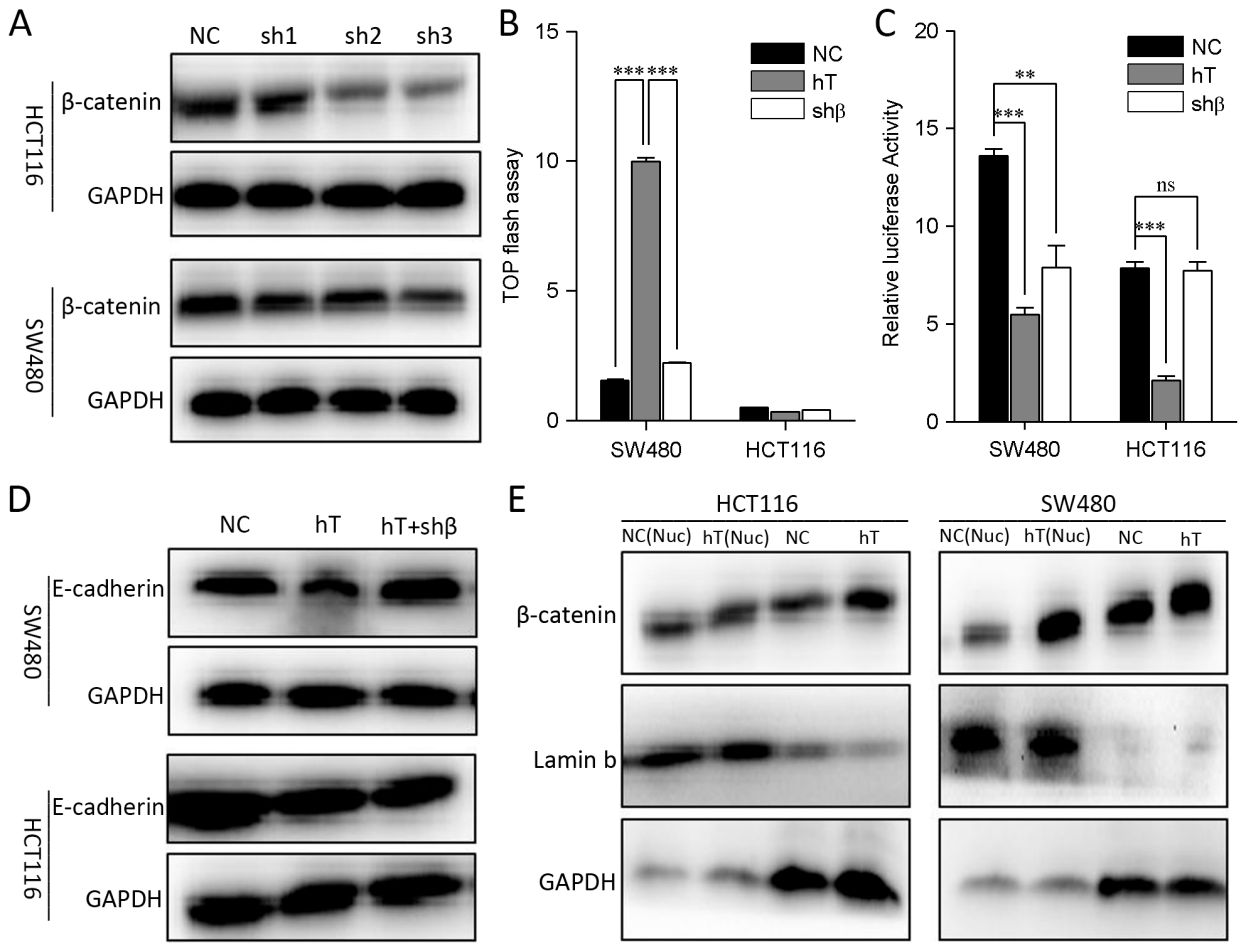
Interfere with β-catenin in HCT116 and SW480 cells **A.** β-catenin knocks down in HCT116 and SW480 cells. **B.** HCT116 and SW480 cells were co-transfected with 100 nM hTERT plasmid and a TOP-FLASH reporter (0.1 ng/well in each 96-well plate), and 100 nM β-catenin interfere plasmid were added TOP-FLASH reporter activation was tested 48 h later. A scrambled construct was used as a negative control (NC). *P < 0.05 compared to control. **C.** The relative luciferase activity of the E-cadherin promoter in HCT116 and SW480 cells. **D.** Western blot of E-cadherin in HCT116 and SW480 cells(Lane1, No treatment; Lane2, hTERT overexpression; Lane3, hTERT overexpression plus β-catenin inhibition). **E.** Western blot to detect the β-catenin expression in nucleu and the total protein of HCT116 and SW480 cells.

We also knocked down the β-catenin in HCT116 and SW480 cells (Figure 3A). TOP Flash study showed that the Wnt activity was significantly down-regulated in SW480 cells but not in HCT116 cells when β-catenin was disturbed (Figure 3B). Similarly, E-cadherin expression was recovered in SW480 cells but not in HCT116 cells when β-catenin was interferenced (Figure 3D). Therefore, hTERT could promote EMT through a novel pathway other than Wnt signaling.

Since E-cadherin can initiate EMT when it is downregulated, inhibited or lost [13]. Therefore, we investigated whether hTERT affects E-cadherin promoter activity. As expected, hTERT expression indeed reduced E-cadherin promoter activity in HCT116 and SW480 cells (Figure 2C and 2D). However, E-cadherin promoter activity was downregulated in SW480 cells but not in HCT116 cells when Wnt signal was disturbed (Figure 2C, 2D and 3C). Next we found that hTERT could promote β-catenin into the nucleus in SW480 cells but not in HCT116 cells when hTERT was overexpressed (Figure 3E). Therefore, hTERT may induce EMT by repressing E-cadherin promoter activity in CRC cells.

### hTERT and ZEB1 form a complex to bind to the E-cadherin promoter

**Figure 4:**
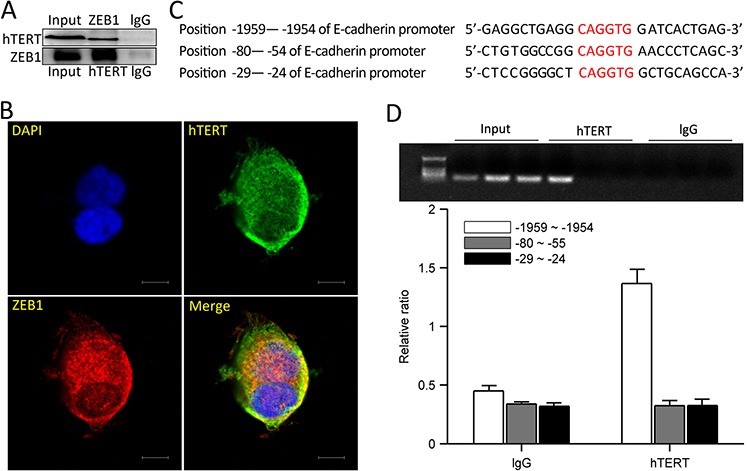
hTERT and ZEB1 form a complex and bind the E-cadherin promoter **A.** HCT116 cells were collected andlysates were subjected to IP using anti-hTERT, anti-ZEB1and rabbit IgG antibodies. Co-precipitating proteins were detected by western blot. **B.** Immunofluorescence staining for hTERT (green) and ZEB1 (red) in HCT116 cells. A representative mergedimage is shown (yellow denotes colocalization). **C.** Three binding sites for the E-cadherin promoter were designed. **D.** PCR quantification of immunoprecipitated E-cadherin promoterin ChIP assays from HCT116 cells with Abs against hTERT and the respective control IgG. Amplified E-cadherin promoter regions contained ZEB1 binding sites at −1959 and −1954. Values represent relative binding to input.

To investigate how hTERT suppresses E-cadherin promoter activity, we used co-immunoprecipitation experiments to determine if hTERT interacts with ZEB1, and we found a direct interaction between them (Figure 4A), confocal imaging also revealed they were co-localized in HCT116 cells (Figure 4B). Next we examined whether hTERT binds to the E-cadherin promoter via ZEB1. We designed three primers that target the ZEB1 binding sites within E-cadherin E-box elements (Figure 4C). We used the hTERT antibody to perform chromatin immunoprecipitation(ChIP) with the E-cadherin promoter and found that hTERT interacted with the E-cadherin promoter within the −1959bp to −1954bp region (Figure 4C and 4D). These data demonstrate that hTERT and ZEB1 form a complex that binds to the E-cadherin promoter within the −1959bp to −1954bp region.

### hTERT promotes EMT through ZEB1 pathway

**Figure 5:**
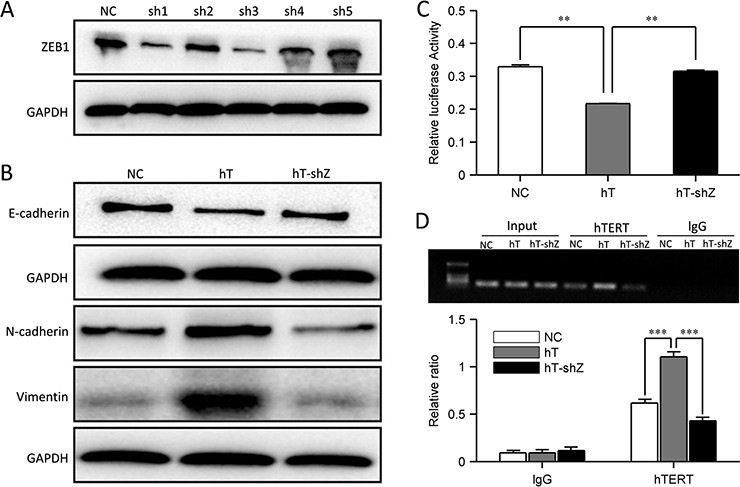
hTERT promotes EMT through the ZEB1 pathway **A.** ZEB1 knocks down in HCT116 cells. **B.** Western blot of E-cadherin, *N*-cadherin and vimentin after hTERT overexpression and ZEB1 knock down in HCT116 cells. **C.** The relative luciferase activity of the E-cadherin promoter in NC-HCT116, hT-HCT116 and shZ-hT-HCT116 cells. **D.** PCR quantification of the E-cadherin promoter at −1959and −1954 immunoprecipitated in ChIP assays from HCT116, hT-HCT116 and shZ-hT-HCT116 cells with Abs against hTERT andthe respective control IgG. Values represent relative binding to input.

To test our hypothesis that hTERT inhibits E-cadherin expression and promotes EMT through the ZEB1 pathway, we constructed ZEB1 RNA interference constructs and confirmed the knockdown by Western blot (Figure 5A). We transfected hT-HCT116 cells with the most efficient shRNA construct. Interestingly, ZEB1 knockdown in hT-HCT116 cells rescued E-cadherin expression (Figure 5B), whereas *N*-cadherin and vimentin expression was decreased (Figure 5B). Immunofluorescence analysis confirmed these results (Supplementary Figure S3A). Therefore, hT-HCT116 cells underwent MET after ZEB1 was knocked down. The cells adopted an epithelial-like morphology, consistent with MET (Supplementary Figure S3B). We next examined E-cadherin promoter activity after ZEB1 knockdown, and found that E-cadherin promoter activity was increased (Figure 5C). ChIP assays showed that hTERT overexpression increased its association with the E-cadherin promoter, and this association was decreased after ZEB1 knockdown (Figure 5D). We also found that ZEB1 was up-regulated in SW480 cells but not in HCT116 cells when overexpressing hTERT (Supplementary Figure S3C). These data suggest that hTERT can restrain E-cadherin promoter activity through binding to ZEB1, and then promote EMT.

### hTERT-ZEB1 interaction promotes tumor migration and metastasis both *in vitro* and *in vivo*

**Figure 6:**
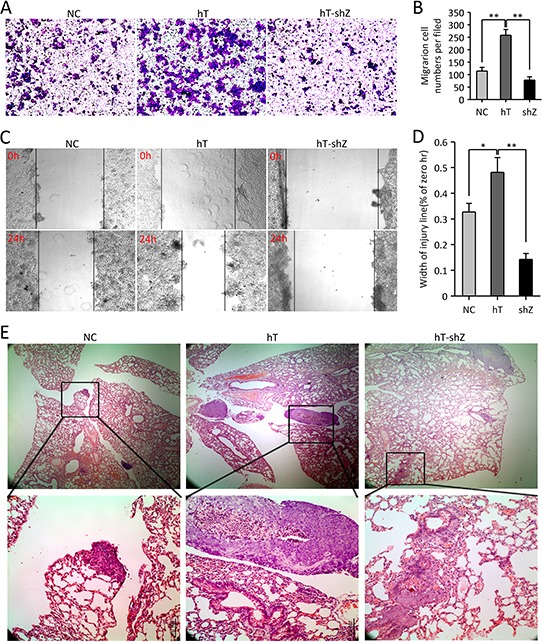
The hTERT/ZEB1 complex promotes the metastatic potential of HCT116 colorectal cancer cells *in vivo* and *in vitro* **A–B.** Cell invasion was measured by Transwell assays 48 h after incubation. The number of cells from three random areas of the membrane was counted using light microscopy; *P < 0.05. **C–D.** The migratory properties of cells were tested in wound healing assays 24 h after incubation. The distance of cells from three random areas of the wound was counted using light microscopy; *P < 0.05. **E.** Metastasis observed after intravenous injection of cells. Metastases were observed by H&E staining at different magnifications.

To examine the biological impact of hTERT and ZEB1 on metastatic of CRC cells, we overexpressed hTERT and silenced ZEB1 in HCT116 cells. Transwell migration assays demonstrated that hTERT overexpression increased invasiveness in HCT116 cells. In contrast, ZEB1 knockdown plus hTERT overexpression decreased cell invasiveness and migration (Figure 6A and 6B). The similar results were observed in wounding experiments (Figure 6C and 6D). Moreover, we injected HCT116 cells, hT-HCT116 cells or hT-shZ-HCT116 cells into the tail vein of nude mice. Lung metastasis was significantly increased when mice were injected with hTERT overexpression, but decreased when injected with hT-shZ-HCT116 cells (Figure 6E). Taken together, the hTERT/ZEB1 complex is associated with cancer invasiveness and metastasis in CRC *in vivo*.

## DISCUSSION

It has been shown that hTERT is highly expressed in most human cancers [18], the induction of hTERT expression and telomerase activation are prerequisites for cellular immortalization and malignancy [19]. However, it has become apparent in recent years that hTERT functions extend beyond telomere maintenance, including DNA-damage repair, RNA-dependent RNA polymerase and Wnt pathway activation [20]. Previous studies of hTERT were related to the infinite proliferation of tumor cells, whereas its function in invasion and metastasis was rarely reported. Clinical studies show that high hTERT expression is associated with poor outcome of various human malignancies [21–24].

EMT, an absolute requirement for tumor invasion and metastasis, plays a key role in cancer progression [25, 26]. Studies have shown that hTERT promotes tumor invasion and metastasis in gastric cancer, liver cancer, and esophageal cancer [24, 27, 28], and that hTERT promotes EMT through the Wnt signaling pathway in gastric cancer and osteosarcoma [8, 15]. However, Wnt signaling is inactivated in some CRC cells [9], in which we found hTERT expression promotes EMT.

Previous studies have shown that ZEB1 plays an important role in regulating E-cadherin expression to tumor invasion and metastasis, and its expression is closely related to the prognosis of cancer patients [10, 29]. ZEB1 is regulated by several factors; include p53, microRNAs, ubiquitin ligase and Wnt [30–33]. ZEB1 can directly bind to the E-cadherin promoter E-box region to inhibit its expression and promote EMT [34]. Another study found that ZEB1 inhibits E-cadherin expression through Brg1 recruitment, and inhibition of the ZEB1-Brg1 interaction can significantly reduce E-cadherin inhibition [14]. We also found that ZEB1 recruits hTERT and binds to E-cadherin promoter to repress its expression.

**Figure 7:**
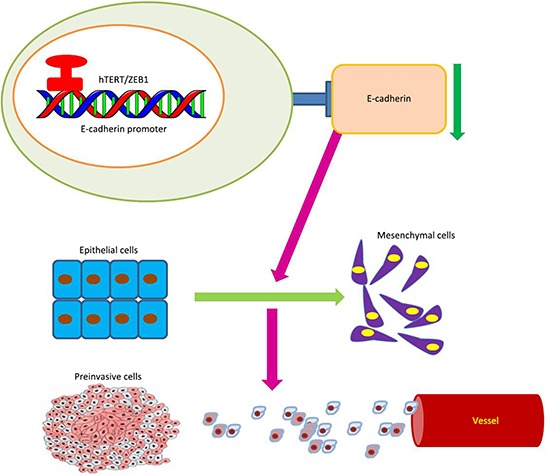
Model of hTERT/ZEB1 complex regulation of E-cadherin expression

We found that the key EMT factor, E-cadherin, is a direct target of the ZEB1/hTERT complex (Figure 7). As a hallmark of the epithelial phenotype, E-cadherin is highly expressed in epithelial cells, but it is repressed when hTERT is over-expressed and EMT is induced in CRC cells. In this study, we found that HCT116 CRC cells without Wnt activity and did not activate Wnt siganling after hTERT was overexpressed. We therefore examined the underlying mechanisms by which hTERT promotes EMT independent of Wnt. As an important moderator, ZEB1 is overexpressed in various cell lines to induce EMT by suppressing E-cadherin [35]. Therefore, we simultaneously overexpressed hTERT and knocked down ZEB1 in HCT116 cells. Interestingly, E-cadherin expression was rescued, suggesting that hTERT regulates E-cadherin expression through ZEB1. By recruiting different co-activators or co-repressors, ZEB1 differentially regulates gene expression [36, 37]. ZEB1 can recruit Brg1 and bind to E-box region of the E-cadherin promoter to repress E-cadherin expression [13]. Thus, we predicted that ZEB1 can also be interacted with hTERT and then binds to the E-cadherin promoter to repress E-cadherin expression. Co-immunoprecipitation and Chromatin immunoprecipitation demonstrated that hTERT interacts with ZEB1 and binds to the −1959 bp to −1954 bp region of the E-cadherin promoter. Both *in vitro* and *in vivo* experiments demonstrated that hTERT promotes tumor invasion and metastasis via interactions with ZEB1. Therefore, it appears evident from the above studies that hTERT and ZEB1 can combine into a protein complex, which binds to E-cadherin promoter region together to inhibit the expression of E-cadherin, and finally contributes to the occurrence of EMT.

In summary, we demonstrate for the first time that E-cadherin is a direct hTERT/ZEB1 target in CRC cells and acts as an effector of this signaling pathway to regulate genes associated with tumor invasiveness. Our findings provide novel insights into hTERT-promoted the occurrence of EMT in CRC. The establishment of such a link establishes hTERT as an important diagnostic predictor and a potential therapeutic target in CRC.

## MATERIALS AND METHODS

### Cell culture and transfection

SW480 and HCT116 cells were obtained from the American Type Culture Collection, cultured in Dulbecco's modified Eagle medium (Hyclone, Logan, UT, USA) and supplemented with 10% FCS (Hyclone, Logan, UT, USA). Cells were transfected with expression, negative control (NC) or reporter vectors using Lipofectamine 2000 (Invitrogen, Carlsbad, CA, USA). Cells were collected 48 h after transfection for western blotting and qRT-PCR analyses. SW480 and HCT116 cells were infected with lentiviral particles encoding the overexpression hTERT plasmid and puromycin resistance. Infected cells were selected through puromycin-containing media.

### Designand construction of shRNA, luciferase reporters and lentiviral constructs

The overexpression hTERT plasmid was packaged into lentiviral particles. hTERT Plasmid (pGRN145, a kind gift from Dr Kevin Kaster, Geron Inc., USA) contained 3.45-kb cDNA between the two EcoRI restriction endo-nuclease sites and was 14-kb long. pIRES2-EGFP purchased from BD Biosciences (Clontech, USA). The hTERT fragment was digested from pGRN145 with EcoRI and ligated into the site of EcoRI of pIRES2-EGFP. The orientation of recombinant DNA was further confirmed by NotI restriction endonuclease. The pIRES2-EGFP as a blank control. The TCF/LEF reporter kit was purchased from SABiosciences (Valencia, CA, USA). To determine the effect of hTERT on TCF/LEF reporter activity, we transfected hTERT-overexpressing or ZEB1 depleted cells with TCF/LEF reporter plasmids. E-cadherin promoters and ZEB1 and β-catenin shRNA plasmids used in this study were designed and synthesized by Guangzhou RiboBio Company (RiboBio, Guangzhou, China).

### RNA extraction, reverse transcription and qPCR

Total cellular RNA was extracted with Trizol ((MRC, Inc, USA), cDNA was synthesized with a PrimeScript RT Reagent Kit (TaKaRa, Ohtsu, Japan) and qPCR was performed with a SYBR Green kit (Takara). The PCR strategy was as follows. Duplicate DNA samples were amplified in parallel 20-μl PCR reactions. For Slug amplification primer was used: (forward, 5′ ATCTGCGGCAAGGCGTTTTCCA 3′, reverse, 5′GAG CCCTCAGATTTGACCTGTC 3′), for Snail amplification of forward and reverse primer was used: (forward, 5′ TGCCCTCAAGATGCACATCCGA 3′, reverse, 5′ GGGA CAGGAGAAGGGCTTCTC 3′), and for Twist amplification of forward and reverse primer was used: (forward, 5′ GCCAGGTACATCGACTTCCTCT3′, reverse, 5′TCCATC CTCCAGACCGAGAAGG 3′). All PCRs were conducted in an Applied Biosystems 7500 real-time PCR machine (Applied Biosystems, Foster City, CA, USA). The thermal cycling profile for both amplicons began with 95°C incubation for 1 min to activate the Platinum Taq DNA polymerase. For the Slug, Snail and Twist PCR, those were followed by 40 cycles of 15 sec at 95°C and 1 min at 65°C. The specificity of all amplifications was determined by melting curve analysis

### Western blot assays

SW480 and HCT116 cells were collected and lysed in RIPA buffer. Aliquots (25 μg) of total or nucleus protein were boiled with RIPA buffer and loaded onto 8% or 10% polyacrylamide gels and transferred to PVDF membrane (Immobilon-P, Millipore). Membranes were blocked for nonspecific antibody binding in 5% nonfat milk and incubated with the corresponding primary and secondary antibodies. GAPDH antibodies were purchased from Santa Cruz Biotechnology Inc. (Santa Cruz, CA, USA). hTERT, ZEB1, E-cadherin, *N*-cadherin and vimentin antibodies were obtained from Epitomics (Abcam, Cambridge, UK).

### Dual-luciferase assay

SW480 and HCT116 cells were seeded in 96-well plates, and cells at approximately 70% confluence were transfected with the pMIR-E-cadherin luciferase reporter. The pRL-TK vector (Promega, Madison, WI, USA), hTERT and NC plasmid were previously described [38]. Cell lysates were harvested within 48 h after transfection. Luciferase activity was detected as the average of three independent assays using a Dual-luciferase assay system (Promega). Firefly luciferase activity was normalized against Renilla luciferase activity.

### Co-immunoprecipitation (Co-IP)

The protein for co-immunoprecipitation were collected using a Universal Magnetic Co-IP Kit (Active Motif, CA, USA). To confirm the interaction of endogenous proteins, lysates were precleared with 25 μL Anti-Rabbit IgG Magnetic Beads (Active Motif) for 1 h at 4°C. The supernatant was discarded, and anti-hTERT and anti-ZEB1 (Abcam) were incubated with the precleared cell lysates for 4 h at 4°C. The Magnetic Anti-Rabbit IgG Bead complexes were washed three times with IP wash buffer (Active Motif) and eluted in 2 × SDS loading buffer, followed by SDS/PAGE and immunoblotting.

### Chromatin immunoprecipitation (ChIP)

ChIP was performed using the MAGNE ChIP A/G kit (Merck Millipore Guyancourt, France) according to the manufacturer's instructions. Briefly, NC-HCT116, hT-HCT116, and shZ-hT-HCT116 cells were incubated for 10 min in 1% formaldehyde at room temperature, followed by 10× Glycine to quench any unreacted formaldehyde. DNA fragments were extracted from lysates using the EZ-Zyme™ Chromatin Prep Kit (Merck Millipore). Mouse anti-hTERT (Abcam) and normal mouse IgG (Abcam) antibodies were used for ChIP. DNA fragments were quantified by PCR as detailed above. Identification of DNA binding sequences for ZEB1 and E-cadherin and PCR primer design was performed in MacVector software. For the ZEB1 binding site at position three E-box region of the human E-cadherin promoter, theprimers used to amplify the region between −29 and −24 were as follows: forward 5′-AACCCAGTGGAATCAGAACCG-3′ and reverse 5′-C AGATACGCTCCGGCCCAC-3′, between −80 and −54 as follows: forward 5′-CTGTGGCCGGCAGGTGAAC 3′ and reverse 5′-GGAGAGTCACCGCAGCCTTGA-3′, between −1959 and −1954 as follows: forward 5′-CCACCACGACTGGCTAATTT-3′ and reverse 5′- TTTAATCGGTCAGCACCACC-3′. All values shown represent relative binding related to the input and are the average of three independent ChIP experiments. PCR of ChIP assays used 1 μL of immunoprecipitated DNA, and PCR products were analyzed on a 1.5% agarose gel by Ethidium Bromide staining.

### Immunofluorescence analysis

HCT116 cells were used for immunofluorescence double labeling studies. Double labeling assays were performed by staining with anti-hTERT and anti-ZEB1(Abcam). Cells were grown on coverslips, fixed with 4% paraformaldehyde, permeabilized in PBS containing 0.5% Triton X-100 for 15 minutes, and then incubated with a non-specific binding blocking solution (5% goat serum plus 4% BSA in PBS) for 30 minutes, and followed incubated with the hTERT and ZEB1 primary antibodies or IgG-matched isotype control antibody overnight at 4°C. Then cells were incubated with the secondary antibodies conjugated to Alexa-Fluor 568 or Alex-Fluor 488 and stained with DAPI. Slides were viewed on a fluorescence microscope.

### Invasion and migration assays

For invasion assays, three days after hTERT overexpression and ZEB1 shRNA treatment, 2 × 10^4^ cells were seeded onto the upper surface of the 24-well Transwell filter inserts (Merck Millipore). After 48 h, cells were fixed in 4% formaldehyde, and cells in the lower chamber were stained with Hoechst33258 (Invitrogen) and quantified by fluorescence microscopy. About 1 × 10^6^ cells were seeded in six-well culture dishes for migration assays, included NC-HCT116, hT-HCT116 and hT-shZ-HCT116 cells. A wound was made in the center of the culture 24 h later, and phase-contrast pictures were acquired at different time intervals. For the experimental metastasis assays *in vivo*, NC-HCT116, hT-HCT116 and hT-shZ-HCT116 cells were injected at a final concentration of 1 × 10^6^ cells/100 μl into the tail veins (i.v.) of female 7 week-old Balb/c nude immunocompromised mice (NOD-SCID). Mice were sacrificed after 30 days, and lung metastasis was quantified.

### Statistical analysis

All data are presented as the mean ± SE, and analyses were performed using Prism 5.0 software (GraphPad, San Diego, CA, USA). The independent-samples test was used to compare two groups, and one-way ANOVA was used to compare three or more groups. *P* < 0.05 was considered statistically significant.

## SUPPLEMENTARY FIGURES



## Abbreviations

CRC: Colorectal cancer
hTERT: human telomerase reverse transcriptase
ZEB1: zinc-finger E-box binding homeobox1
EMT: Epithelial-to-mesenchymal transition
MET: Mesenchymal-to-epithelial transition
TCF-LEF: T-cell and lymphoid enhancer
BRG1: Brahma-related gene1
Co-IP: Co-Immunoprecipitation
ChIP: Chromatin Immunoprecipitation.
