# Supplementary material for: An hTERT/ZEB1 complex directly regulates E-cadherin to promote epithelial-to-mesenchymal transition (EMT) in colorectal cancer
Source: Oncotarget. 2015 Oct 20;7(1):351–61. doi: 10.18632/oncotarget.5968 (PMC4808003; doi:10.18632/oncotarget.5968)
Supplement: Supplementary file 1 [file oncotarget-07-0351-s001.pdf]

SUPPLEMENTARY FIGURES

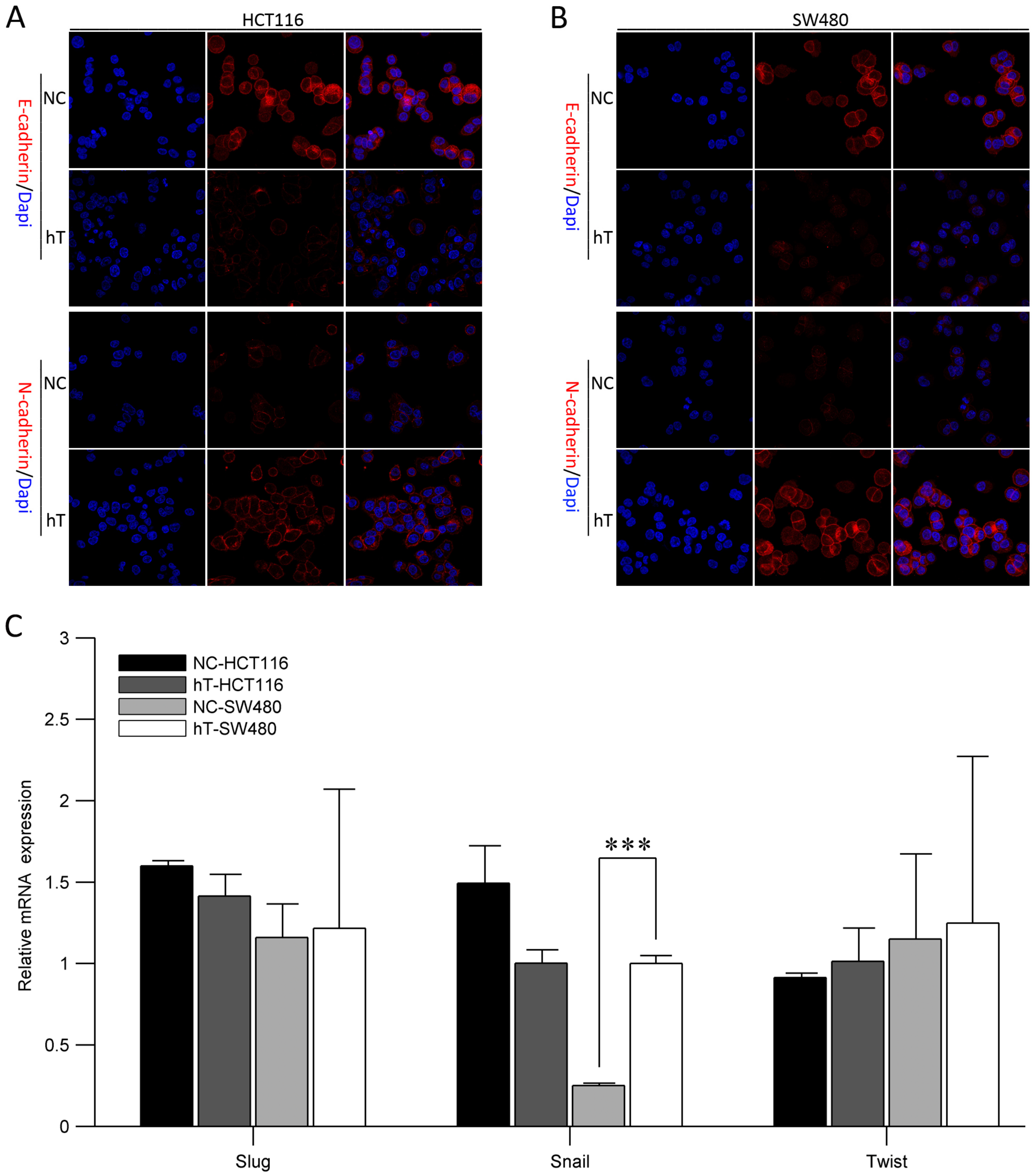

Supplementary Figure S1: A–B. Immunofluorescence of E-cadherin and *N*-cadherin in SW480 and HCT116 cells after hTERT overexpression. **C.** qRT–PCR of Slug, Snail and Twist in SW480 and HCT116 cells after hTERT overexpression.

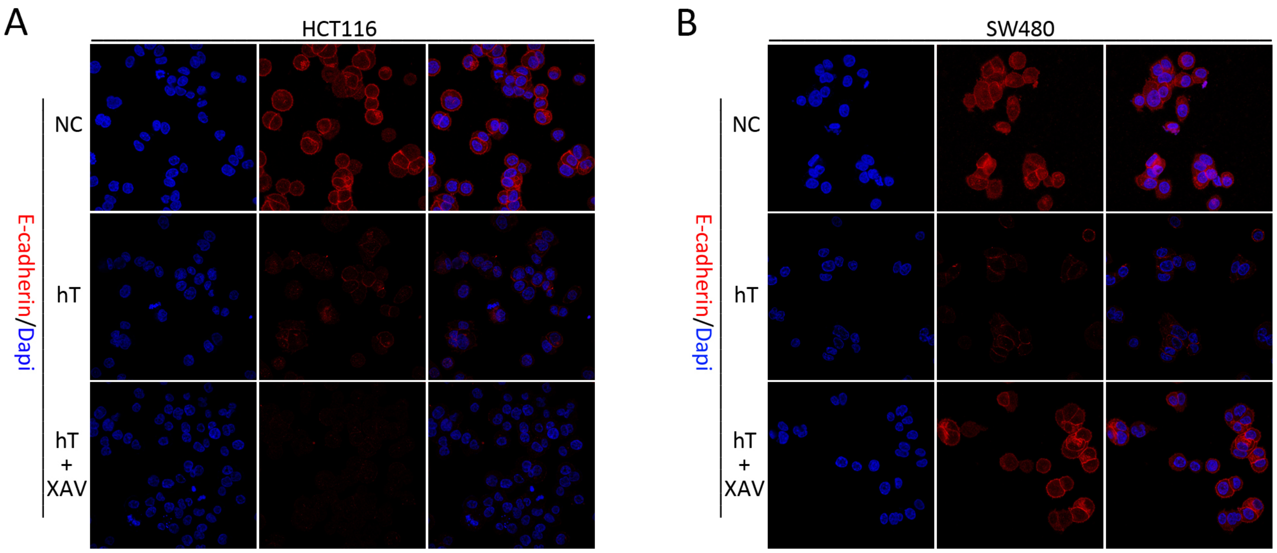

Supplementary Figure S2: A–B. Immunofluorescence of E-cadherin in SW480 and HCT116 cells after hTERT overexpression and Wnt inhibition (XAV).

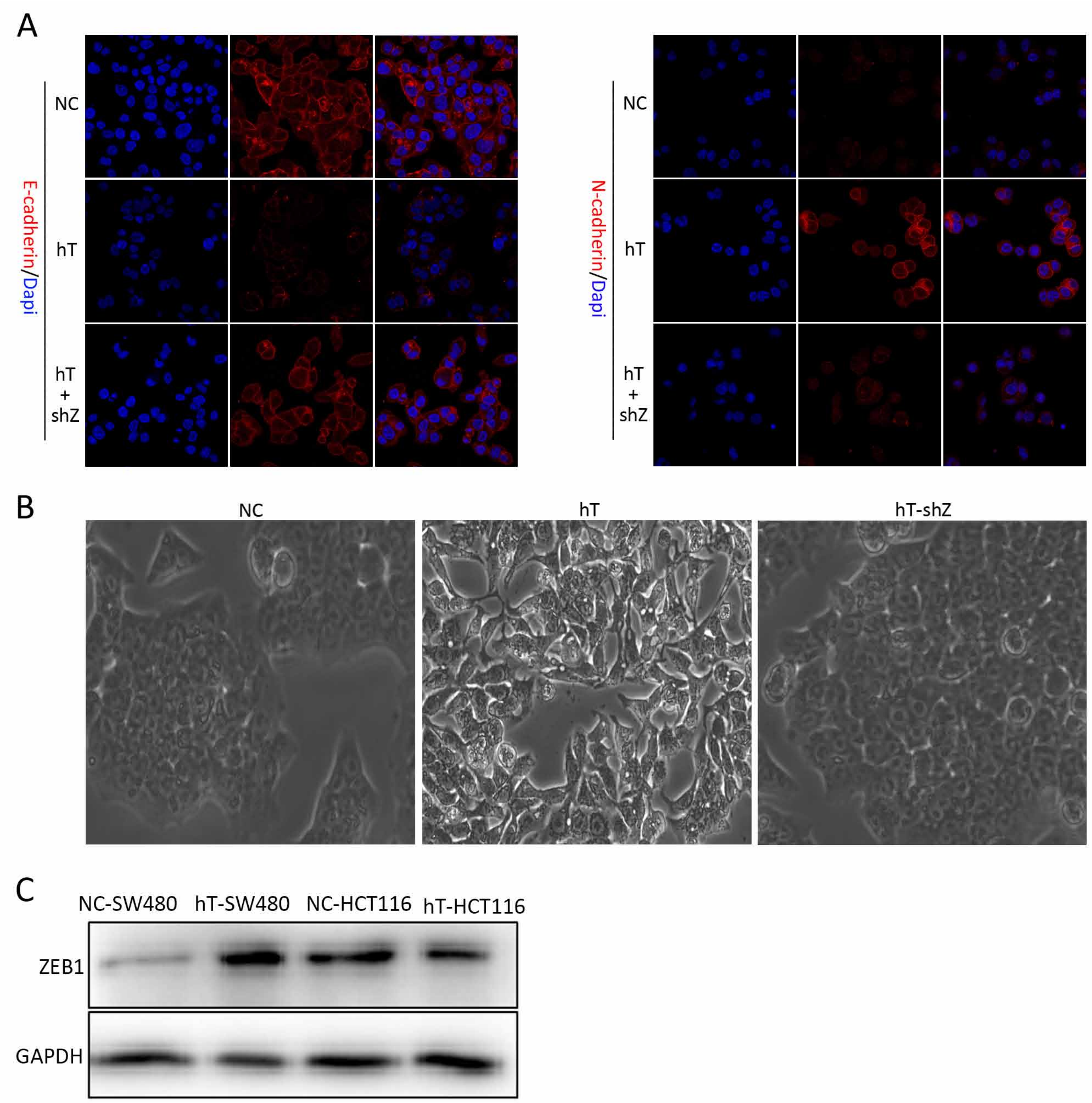

Supplementary Figure S3: A. Immunofluorescence of E-cadherin in HCT116 cells after hTERT overexpression and ZEB1 knock down. **B.** Cell morphological changes after hTERT overexpression and ZEB1 knock down in HCT116 cells. **C.** ZEB1 expression was analyzed by western blot after hTERT overexpression in SW480 and HCT116 cells.
